# Knowledge and Attitudes Toward Strep Throat and Rheumatic Fever: A Cross-Sectional Study Among the United Arab Emirates Population

**DOI:** 10.1055/s-0046-1822816

**Published:** 2026-06-03

**Authors:** Ahmad Hisham Al-Anoud, Mawiah Zhour Adi, Bayan Bozalasal, Saryia Adra, Kifaya Tamimi, Laila Nour Aldeen Ismail, Hiba Jawdat Barqawi

**Affiliations:** 1 Department of Clinical Sciences, College of Medicine, University of Sharjah, Sharjah, United Arab Emirates; 2 Department of Internal Medicine, Harlem Hospital Center, Columbia University, New York, United States; 3 Department of Obstetrics and Gynaecology, Dubai Health Authority, Dubai, United Arab Emirates; 4 Research Institute of Medical and Health Sciences, University of Sharjah, Sharjah, United Arab Emirates

**Keywords:** strep throat, rheumatic fever, rheumatic heart disease, public knowledge, antibiotic use, UAE

## Abstract

**Background:**

Group A Streptococcus pharyngitis, or strep throat, can lead to rheumatic fever (RF) and rheumatic heart disease (RHD) if left untreated. While these conditions are a public health concern, limited evidence exists on public awareness in the United Arab Emirates (UAE). This study assessed the knowledge and attitudes toward strep throat and RF among UAE residents.

**Methods:**

A cross-sectional online survey was conducted between September and November 2024 among adults aged ≥ 18 years. A 28-item questionnaire was distributed via social media platforms. Data were processed using SPSS, with bivariate and multivariate analyses applied to identify factors associated with knowledge levels.

**Results:**

Among the 462 respondents, 64.9% were female, and over half held a bachelor's degree or higher. Misconceptions were common: 47.4% believed cold water causes sore throat, and 53.7% reported using antibiotics for symptom relief, often without physician advice. Note that 60.6% had never heard of strep throat, and only 39.0% recognized its association with RF. While most participants identified joint pain as a symptom of RF, fewer recognized heart complications as a major consequence. The median knowledge score was –1 (interquartile range = 5), and none answered all questions correctly. Females, health care workers, and participants with comorbidities demonstrated significantly higher knowledge scores ( *p* = 0.005, *p* < 0.001, and *p* = 0.049, respectively).

**Conclusion:**

The study revealed limited public knowledge of strep throat and RF in the UAE, alongside inappropriate antibiotic use. Targeted awareness campaigns are urgently needed to address misconceptions, encourage appropriate health care-seeking behavior, and reduce the burden of RF and RHD.

## Introduction

Throat infections are common and may be caused by viruses or bacteria, with most being mild and self-limiting. Strep throat is a bacterial infection caused by Group A Streptococcus ( *Streptococcus pyogenes* ) that requires proper treatment to prevent complications such as rheumatic fever (RF). 1 Strep throat and RF are a public health priority for the majority of low- to middle-income countries. Despite the United Arab Emirates (UAE) being classified as a high-income country, it has a diverse population, with many expatriates coming from low- and middle-income countries where RF and rheumatic heart disease (RHD) remain prevalent. The persistence of these conditions in the UAE may be attributed to this demographic makeup as well as possible differences in health awareness, access to care, and health care-seeking behavior. RF is an autoimmune reaction resulting from untreated Group A Streptococcus pharyngitis that evolves into RHD, where valvular involvement is permanent and results in multiple adverse cardiovascular events including heart failure, arrhythmias, and premature death. 2 The disease is rare in areas with sufficient access to health care, showing a decrease in the incidence of RF especially in developed countries, which is attributed to the improved living conditions, use of antibiotics, and possibly the shifting of Group A Streptococcus serotypes, whereas it continues to be a major cause of morbidity and mortality among young people in developing nations. 3 Per the World Health Organization, RHD has a mortality over 300,000 people yearly and affects around 40 million people worldwide. 4 However, to date there is no data quantifying RF and RHD burden in the UAE.

RF highlights gaps in awareness, late diagnosis, and suboptimal prophylactic antibiotic use, all of which are preventable. Globally, not many studies were conducted regarding knowledge and attitudes toward RF and RHD, and those that did were among health care workers. 5 6 Furthermore, in the Middle East and North Africa (MENA) region, only a few studies assessed the knowledge and attitudes of the general population toward sore throat and RF. 7 8 To our knowledge, to date, there have been no comprehensive studies assessing the public's knowledge and attitudes toward RF in the UAE. In the diverse population of the UAE, there are disparities in access to health care, highlighting the importance of raising awareness regarding diseases among the population to ensure better public health outcomes. Early diagnosis and effective treatment of Group A Streptococcus could subsequently lead to a substantial decline in RF incidence in the region. Moreover, an informed public that recognizes the symptoms and acts by seeking early treatment should be policy makers' priority, 9 highlighting the urgency of commencing public health initiatives, aiming to create public awareness and promote early intervention.

The aim of this project was to evaluate the attitudes and awareness related to sore throat and RF among the general population of the UAE. The findings could provide valuable insights into determining the need for public health preventative measures, thus reducing the burden of RF in the UAE.

## Methodology

### Study Design and Target Population

An observational, cross-sectional study was conducted to investigate the awareness and attitudes of the general population in the UAE toward sore throat and RFs. Our study population included UAE residents, aged 18 years and above, who spoke either English or Arabic. Those below 18 years of age, nonresidents/tourists, and those without access to social media platforms were excluded from the study. The calculated minimum study sample size was 385, based on a 5% margin of error and a 50% prevalence using *n* = 4p (1 - p)/SE ^2^ , where *n* is the sample size, *p* is the expected prevalence, and *SE* is the sampling error. To account for nonresponse, attrition, and incomplete responses, the sample size was increased by 20%, to 462 minimum sample size. This research was approved by the Research Ethics Committee of the University of Sharjah (Reference Number: REC-24-06-07-01-S).

### Data Collection Instrument and Procedure

A questionnaire was adapted from previous studies. 7 9 10 The 28-item questionnaire was divided into three parts: demographics (10 questions), knowledge (13 questions), and attitudes (5 questions). The questionnaire was drafted in English, and then translated to Arabic. The Arabic questionnaire was reviewed multiple times to ensure consistency with the English version. Both versions were pilot tested (four times each); all provided feedback was evaluated and incorporated if appropriate, before finalizing the questionnaire.

Before beginning the questionnaire, an information sheet outlining purpose, voluntary participation, and confidentiality of response was presented to the potential participants. Their voluntary response to the first question in the questionnaire, regarding their willingness to complete the survey, was considered written consent to participate in the study. The questionnaire was hosted on Google Forms and distributed through different social media platforms such as: WhatsApp, Instagram, Facebook, LinkedIn, and email. Data collection took place between September 19 and November 30, 2024. Finally, the collected data, which does not contain any identifying information, was stored securely and accessible only by the investigators to ensure confidentiality.

### Analysis of Data

Data was exported from Google Forms to Microsoft Excel for data cleaning. It was then further analyzed on SPSS Statistics for Windows, version 26.0 (IBM Corp., Armonk, New York, United States). Continuous variables were assessed for normality using histograms, Q-Q plots, and the Shapiro–Wilk test. The median and interquartile range (IQR) was reported for nonnormally distributed continuous variables and valid percentages were acknowledged for any missing responses. Association between demographic characteristics with knowledge and attitude levels was tested using bivariate analysis. Statistical tests used to test the association included Mann–Whitney *U* , Kruskal–Wallis, and chi-square tests for continuous and categorical variables, respectively. Statistical significance was set at *p* -value ≤ 0.05.

## Results

A total of 484 responses were collected, out of which 22 were discarded as they did not meet the inclusion criteria; hence, 462 responses were analyzed. Less than a third (29.2%, *n* = 187) of the participants were 25 years old or younger. Females constituted about 64.9% ( *n* = 300) of the participants, 61.3% ( *n* = 283) were married, and 59.5% ( *n* = 275) had children. The fields of work varied, with 44.4% ( *n* = 205) working in non-health care sectors. Lastly, 69.0% ( *n* = 319) of participants reported having health insurance. Further demographic details are provided in Table 1 .

**Table 1:** Demographics of the study's participants

Characteristic	Frequency ( *n* )	Percent (%)	Characteristic	Frequency ( *n* )	Percent (%)
Sex			Place of residence		
Male	162	35.1	Abu Dhabi	43	9.3
Female	300	64.9	Dubai	123	26.6
Age			Sharjah	212	45.9
18–25	135	29.2	Ajman	53	11.5
26–45	178	38.5	Other Emirates	31	6.7
46–65	149	32.3	Field of work		
Highest degree obtained			Health care	79	17.1
High school or lower	103	22.3	Non-health care	205	44.4
Diploma	32	6.9	Student - health care	43	9.3
Bachelor's Degree	239	51.7	Student - non-health care	59	12.8
Postgraduate Degree	88	19.0	Housewife	76	16.5
Marital status			Do you have health insurance?		
Not Married	179	38.7	No	143	31
Married	283	61.3	Yes	319	69
Do you have children?			Do you have any comorbidity?		
No	187	40.5	No	390	84.4
Yes	275	59.5	Yes	72	15.6
Nationality					
UAE	69	14.9			
Other Arab	321	69.5			
Non-Arab	72	15.6			

Abbreviation: UAE, United Arab Emirates.

**Fig. 1:**
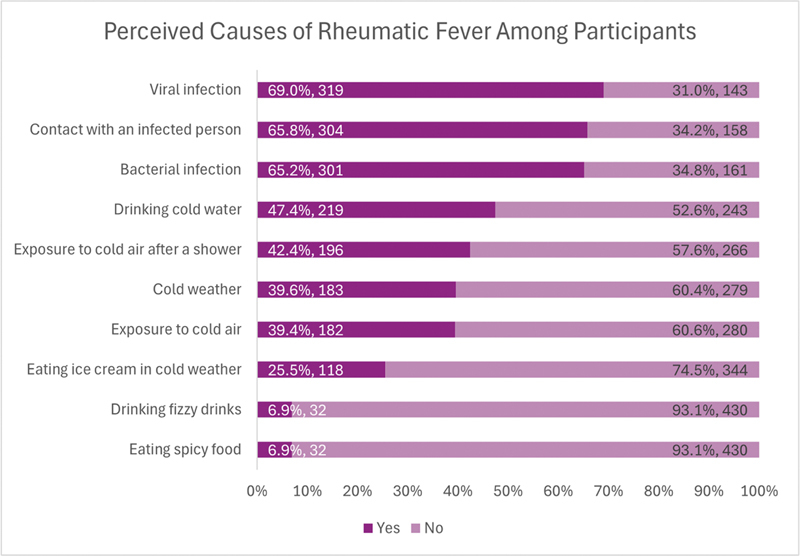
Perceived causes of rheumatic fever among participants.

When asked about causes of sore throat, 47.4% ( *n* = 219) incorrectly identified drinking cold water as a cause. Note that 39.4% ( *n* = 182) thought that cold air drafts and 42.4% ( *n* = 196) thought that being exposed to cold air after a shower causes sore throat ( Fig. 1 ). Additionally, 69.0% ( *n* = 319) and 65.2% ( *n* = 301) believed that viral and bacterial infections are the underlying causes of sore throat. When asked what do the participants use to relieve sore throat symptoms, 53.7% ( *n* = 248) admitted to using antibiotics, while 5% ( *n* = 23) do not use anything. Many participants, however, utilized herbal tea (61.9%, *n* = 286), honey (47.6%, *n* = 220), and Acetaminophen (38.7%, *n* = 179). As for sources of knowledge, 77.7% ( *n* = 359) identified doctors, followed by 59.5% ( *n* = 275) for family and 45.9% ( *n* = 212) for pharmacists ( Supplementary Fig. S1 , available in the online version only). Among those who used antibiotics for sore throat, 43.7% ( *n* = 45/103) stated that it was following nondoctors' advice, while 56.5% ( *n* = 203/359) were following a physician's advice. Interestingly, individuals who consulted doctors were 1.49 (95% confidence interval: 1.06–2.11, *p* = 0.025) times more likely to take antibiotics compared to those who relied on advice from nondoctors. When participants were asked about the circumstances under which they seek medical attention, the majority, 68.6% ( *n* = 317), indicated they would do so if sore throat persisted for more than a few days, while 52.8% ( *n* = 244) seek care if they had a fever. In contrast, only 8.4% ( *n* = 39) reported seeking medical attention immediately.

The median knowledge score of participants when it came to strep throat and RF was –1 (IQR = 5) on a scale from –36 (lowest possible score) to 36 (highest possible score), the participants' score ranged from –11 to 11. Surprisingly, none of the participants answered all knowledge items correctly ( Supplementary Fig. S2 , available in the online version only). Multiple logistic regression revealed that the knowledge score was significantly affected by the participants' sex, field of work, and presence of comorbidities ( Table 2 ). Females, those in health care fields, and those with comorbidities had higher knowledge score ( *p* = 0.005, *p* < 0.001, and *p* = 0.049, respectively).

**Table 2:** Factors affecting the knowledge score among the participants

Parameter	Knowledge score		
	Odds ratio	95% CI	*p* -Value
Sex			
Male	Reference		
Female	1.815	1.199–2.748	**0.005**
Nationality			
Non-Arab	Reference		
Arab	0.722	0.417–1.248	0.243
Field of work			
Non-health care	Reference		
Health care	3.175	2.000–5.042	**< 0.001**
Comorbidity			
No	Reference		
Yes	1.712	1.003–2.922	**0.049**

Abbreviation: CI, confidence interval. Significant *p* -Values are bold.

When asked about strep throat, 60.6% ( *n* = 280) never heard about the disease. Interestingly, of those who had heard of it, 68.7% ( *n* = 125/182) correctly identified it as being caused by a bacterial infection. As for mode of transmission, 67.5% ( *n* = 312) correctly identified coughing or sneezing and sharing utensils (43.1%, *n* = 199). Whereas 26.0% ( *n* = 120) thought that merely shaking hands leads to being infected with strep throat. Most of the participants (86.6%, *n* = 400) believed that antibiotics should only be stopped after completing the full course as prescribed by a physician. In contrast, 10.2% ( *n* = 47) said they would stop once their symptoms disappeared, and 3.2% ( *n* = 15) would stop when their temperature normalizes. As for complications of untreated strep throat, most of the participants thought that breathing difficulties (74.0%, *n* = 342) and joint pain (48.7%, *n* = 225) are major complications whereas only 32.9% ( *n* = 152) identified heart problems as a complication and even less thought of kidney issues (19.3%, *n* = 89).

Only 39.0% ( *n* = 180) of participants believed that strep throat is linked to RF. Participants were then asked about symptoms of RF, 67.1% ( *n* = 310) correctly identified joint pain, while skin nodules were identified by only 16.7% ( *n* = 77) and 64.5% ( *n* = 298) incorrectly thought that fever was a symptom. As for the most vulnerable age group, 43.1% ( *n* = 199) correctly identified 5 to 15 years of age, while 38.3% ( *n* = 177) thought that those above 15 years are at the greatest risk of RF. Interestingly, 46.1% ( *n* = 213) thought that RF develops within 1 to 5 days of sore throat symptoms. Finally, 45.5% ( *n* = 210) agreed that it is necessary to treat sore throat with antibiotics and 51.5% ( *n* = 238) agreed that everyone who suffers from sore throat should consult a doctor.

## Discussion

Over the past few decades, the worldwide burden of RF and RHD has declined, likely driven more by socioeconomic progress than by direct disease control measures. 11 It estimated that at least 18.1 million people were affected by invasive *S. pyogenes* infections, with 1.78 million new cases occurring annually. 12 Beyond its health impact, RHD disproportionately affects the world's most vulnerable populations, with the poorest and most marginalized groups at regional, national, and local levels continuing to experience high mortality without significant improvement. 13 Between the year 1990 and 2021, the number of RHD cases in the general population increased significantly from 32.3 to 54.8 million, leading to approximately 200,000 to 250,000 premature deaths annually, with the highest burden observed in Africa, South Asia, and the Pacific Islands. 14 Given these health and economic consequences, it is critical to assess public knowledge and attitudes regarding strep throat and RF. This study was therefore conducted to evaluate awareness among the UAE population, with the aim of identifying knowledge gaps that could guide targeted education and prevention strategies using a scale that was adapted and improved from previous studies conducted in Saudi Arabia. 7 10 Our findings showed a good understanding regarding sore throat and its cause; however, there was a lack of knowledge about strep throat and RF, including its treatment, showing an evident gap in the public knowledge and attitudes.

A major misconception identified in this study is the cause of sore throat. Nearly half of the study's participants identified drinking cold water and being exposed to cold air after shower under the causes of sore throat; this misconception was also observed in one-third of the participants in a Saudi Arabian study. 15 Additionally, while more than two-thirds correctly identified viral infection as the most common cause of sore throat, two-thirds also believed bacterial infections to also be a common cause. Correspondingly, in two studies conducted in different regions in Saudi Arabia, one-third of participants identified both viral and bacterial as causative agents of sore throat. 7 8 This shows a lack of knowledge within the MENA region as the majority of sore throats are caused by viral infections. 16 Furthermore, participants in our study working in the health care field demonstrated a higher knowledge score in regards to sore throat and RF highlighting the importance of the role of health care professionals in spreading awareness and providing educational sessions to the public to correct these misconceptions, thus protecting the public and their children from sore throat, which is considered a risk factor for RF and its complications. 2

Our study showed that 68.6% of the participants would seek medical attention after a few days of persistent sore throat; however, immediate consultation was rare (8.4%). Furthermore, about half of the participants reported using antibiotics for symptom relief, out of which 43.7% used the antibiotics without physician guidance, showing a clear overreliance on antibiotics, coupled with delayed health care-seeking behavior, which may contribute to both inappropriate antibiotic use and inadequate treatment of Group A Streptococcus, thereby increasing the risk of RF. Similar patterns were observed in regional studies: community-based research from Saudi Arabia noted that almost half of the participants would use antibiotics without prescriptions and widespread belief in antibiotics efficacy for sore throat. 17 Internationally, patterns of self-medication and delayed care-seeking for sore throat are also well documented; a large multicountry survey on self-management and nonprescription antibiotic use recorded great variations among countries; from low percentages in some European countries to far greater proportions in parts of the Middle East. 18 This shows that such documented behaviors in the UAE are not confined to low- and middle-income countries, but can exist elsewhere too.

Antibiotic misuse also emerged as a significant concern in our study, with approximately half of the respondents reporting the use of antibiotics for sore throat, while only a small fraction indicated they would avoid taking any medication. This widespread use of antibiotics warrants urgent attention. A study conducted in Nigeria among health care workers found that approximately 85% believed all children with sore throat should be treated with antibiotics. 5 Furthermore, a study conducted among the general population in Saudi Arabia found nearly half of the participants believed antibiotics are helpful in treating sore throat. 15 While this shows basic awareness that bacterial infections require antibiotics, it also portrays a worrying trend of overprescription or self-medication especially in places where use of antibiotics is readily accessible over the counter, 19 which in turn can lead to the rise of resistant bacterial pathogens. 20 Addressing this issue requires joint efforts, particularly from governments and policy makers. Effective antibiotic stewardship programs must be implemented and enforced at a national level to regulate prescribing practices and monitor antibiotic use. For instance, Sweden launched its Strama program in the mid-1990s to coordinate surveillance of antibiotic use and resistance across healthcare levels. Within a decade, Sweden achieved a noticeable decline in antibiotic prescriptions and slowed the rise of antibiotic resistance. 21 Similarly, in the Netherlands, public attitudes toward antibiotics have traditionally been cautious, with many people understanding that sore throats are usually viral and do not require antibiotics. This mindset helps keep inappropriate antibiotic use low and supports national stewardship goals to promote public awareness. 22 Government led policies, such as restricting nonprescriptible antibiotic sales and educating both health care providers and the public, have been shown to significantly reduce misuse. 23 The World Health Organization emphasizes the importance of national action plans in tackling antimicrobial resistance, advocating for legislation, surveillance, and education as core components of stewardship strategies. 24 In the absence of strong government action, efforts by individual healthcare providers and hospitals may be inadequate to address the rising threat of antibiotic resistance.

Our findings show that around 40% of participants agreed that there is an association between strep throat and RF. More than half of the participants of a Saudi study, agreed on the association between sore throat and RF. 7 Another study conducted in Saudi Arabia, found that although 77% of respondents reported a history of sore throat, only 40.5% recognized its potential association to RF. 25 Comparatively, awareness levels were even lower among parents in another region of Saudi Arabia, where only 4.8% demonstrated good knowledge of RF and RHD, while over 70% showed poor understanding. 8 The aggregation of those findings suggests varying regional awareness. Moreover, two-thirds of the participants of our study had moderate knowledge recognizing that strep throat is a bacterial infection with the majority emphasizing on the need to start antibiotics. This trend may stem from the widespread belief that most infections require antibiotics. 26

Furthermore, females over 26 years of age and those with preexisting conditions had a better understanding of RF. A Saudi study also found that females had greater knowledge. 7 This may be because, in this region, women often spend more time with their children and become more familiar with their symptoms and health. 19 20 Such findings shed the light on the importance of targeting awareness campaigns not only to mothers but also to males and younger age groups, to close the knowledge gap and ensure that all caregivers are equally prepared to identify and appropriately manage RF.

Approximately three-fourths of participants in our study believed that breathing difficulties were the most common complication of RF, whereas only one-third knew that untreated RF can lead to heart problems. This suggests that many individuals might underestimate the risk of more serious outcomes such as heart disease, highlighting a gap in awareness that needs to be addressed through targeted health education. Additionally, two studies conducted in Saudi Arabia showed that around two-thirds of their participants recognized heart problems as one of the major complications. 7 10 This highlights the importance of educational campaigns with tailored interventions to address the limited knowledge toward RF and to lower the disease's burden. 21

## Limitations of the Study

Limitations of this study include the use of nonprobability convenience sampling method, resulting in selection bias. Hence, findings may not accurately represent the target population as a whole. It cannot be concluded whether level of knowledge had led to more positive attitudes, and no cause-and-effect relationships can be determined—merely if an association exists. Therefore, future longitudinal studies may help address this gap.

In addition, the use of online platforms for data collection may further limit the generalizability of findings. Participants with greater access to digital technologies and higher digital literacy were more likely to be included, potentially excluding participants with limited Internet access, particularly older adults and those from lower socioeconomical backgrounds. This may have introduced a degree of selection bias, as digital access is frequently linked to higher levels of education and health literacy, which could influence participants' knowledge and attitudes toward strep throat and RF.

## Conclusion

An overall lack of knowledge was found among the general population of the UAE regarding RF and RHD. This knowledge gap might have a role in delaying the diagnosis of these two conditions, and it might also contribute to inadequate preventive measures, which may lead to an increase in their prevalence among the population. Our results can be used to initiate public health measures that aim to educate the UAE community about strep throat, RF, RHD, and the relationship between these conditions.

## Notes

**What We Already Know**

- Rheumatic fever and rheumatic heart disease remain significant public health concerns in many low- and middle-income countries, despite being controlled in high-income regions.
- Inadequate knowledge and awareness regarding strep throat and its complications often lead to delayed health care seeking and misuse of antibiotics.

**What This Article Adds**

- This study provides the first comprehensive assessment of public knowledge and attitudes toward strep throat and rheumatic fever in the United Arab Emirates.
- The findings identify specific knowledge gaps and misconceptions that can guide future public health education and awareness campaigns aimed at preventing RF and RHD.
- By highlighting patterns of health care-seeking behavior and antibiotic use, this study supports the development of targeted interventions to promote appropriate management of sore throat and prevent avoidable complications.
