# Supplementary material for: Knowledge and Attitudes Toward Strep Throat and Rheumatic Fever: A Cross-Sectional Study Among the United Arab Emirates Population
Source: Avicenna J Med. 2026 Jun 3;16(2):70–6. doi: 10.1055/s-0046-1822816 (PMC13354506; doi:10.1055/s-0046-1822816)
Supplement: Supplementary file 1 — Supplementary Material [file 10-1055-s-0046-1822816-s250162.pdf]

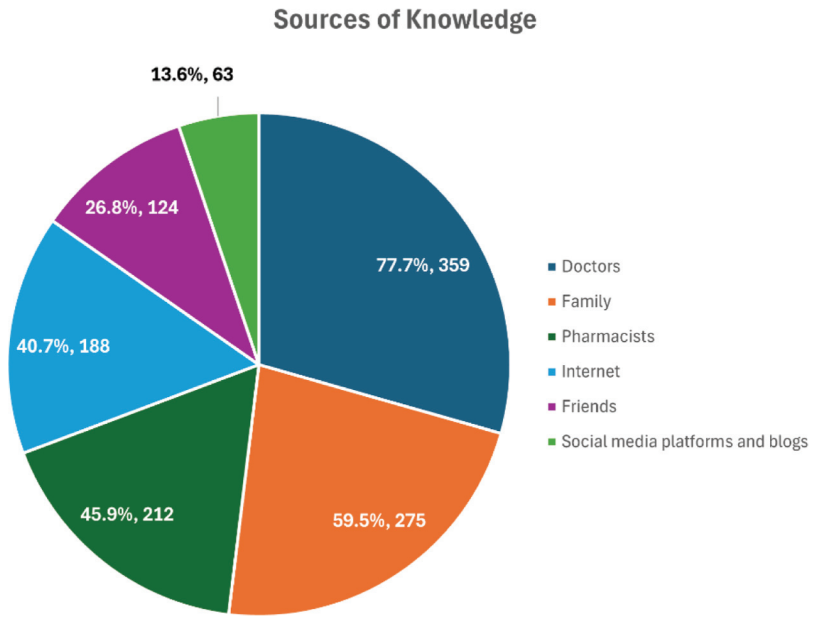

Supplementary Fig. S1 Sources of information used by participants.

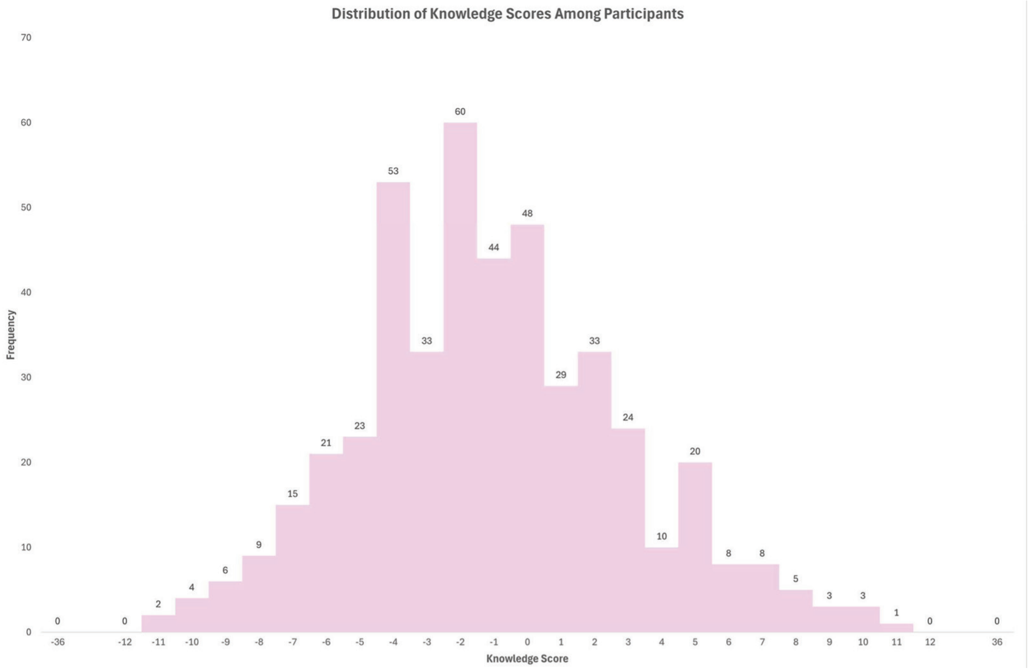

Supplementary Fig. S2 Distribution of knowledge scores among participants.

 © 2026. The Author(s).
